# Correlation dynamics of nitrogen vacancy centers located in crystal cavities

**DOI:** 10.1038/s41598-020-73697-7

**Published:** 2020-10-06

**Authors:** Abdel-Haleem Abdel-Aty, Heba Kadry, A. -B. A. Mohamed, Hichem Eleuch

**Affiliations:** 1 Department of Physics, College of Sciences, University of Bisha, P.O. Box 344, Bisha, 61922 Saudi Arabia; 2 Physics Department, Faculty of Science, Al-Azhar University, Assiut, 71524 Egypt; 3 Department of Mathematics and Computer Science, Faculty of Science, Sohag University, Sohag, Egypt; 4 Department of Mathematics, College of Science and Humanities in Al-Aflaj, Prince Sattam bin Abdulaziz University, Al-Aflaj, 710-11912 Saudi Arabia; 5 Department of Mathematics, Faculty of Science, Assiut University, Assiut, Egypt; 6 Department of Applied Physics and Astronomy, University of Sharjah, Sharjah, United Arab Emirates; 7 Department of Applied Science and Mathematics, College of Arts and Sciences, Abu Dhabi University, Abu Dhabi, United Arab Emirates; 8 Institute for Quantum Science and Engineering, Texas A&M University, College Station, TX 77843 USA

**Keywords:** Quantum information, Qubits, Theoretical physics

## Abstract

In this contribution, we investigate the bipartite non-classical correlations (NCCs) of a system formed by two nitrogen-vacancy (N-V) centers placed in two spatially separated single-mode nanocavities inside a planar photonic crystal (PC). The physical system is mathematically modeled by time-dependent Schrödinger equation and analytically solved. The bipartite correlations of the two N-V centers and the two-mode cavity have been analyzed by skew information, log-negativity, and Bell function quantifiers. We explore the effects of the coupling strength between the N-V-centers and the cavity fields as well as the cavity-cavity hopping constant and the decay rate on the generated correlation dynamics. Under some specific parameter values, a large amount of quantum correlations is obtained. This shows the possibility to control the dynamics of the correlations for the NV-centers and the cavity fields.

## Introduction

The recent years witnessed huge progress towards the production of the quantum devices for different applications in industry and research^1–8^. The quantum computer is one of these devices^9–12^. It was shown that several algorithms and hard protocols, which not possible to be computed using the classical computers, can be solved by the quantum computer^13–18^.

Different challenges have to be resolved to produce an efficient quantum computer for the quantum control, optimization and others. One of the challenges that faced the scientists is how to store the quantum data produced from the quantum operations. Several proposals were suggested to design the quantum memory, for example, nanoresonators, quantum dot, Silicon Dot materials SD and, Nitrogen Vacancies in Diamond NVD^4,19–22^. The NVD has very good physical features (stability, long decoherence time and great optical band gap) which has been recommended as one of the best proposals for the quantum storage^23,24^. The first time of preparing the defects in diamond was in 1997 by Gruber et al.^25^. Based on this experiment, several applications of the defects on diamond were introduced, which make the NVD the most appropriate for quantum information technology industries and quantum memory, for example, implementation of a photostable single photon laser source^26^, optical quantum networks and implementation of optical preparation and readout of the defects as an electronic spin in quantum materials^27,28^. The NV defects have been employed in room temperature demonstrations of quantum registers built upon the NV electronic spin and proximal to N and $^{13}$C nuclear spins. These realizations are considered the first step towards the production of the quantum storage for commercial use^29^. Another experiment supporting the use of the NVD as quantum memory was conducted by Neumann et al. They were able to create high-quality quantum register spins of nitrogen defects in diamond^30^. The theoretical investigation of quantum features of N-V centers, using density functional theory techniques DFTT, (*ab initio* and Gaussian versions) have proven their appropriateness for the quantum storage^31–33^.

Non-classical correlations (NCCs)^34–39^ are the main source for the quantum technology, such as quantum communications^40,41^, dense coding^42^ and quantum, security, and cryptography^43^. Correlations can be categorized into two parts: classical correlations and quantum correlations^44^. There are several quantifiers for the non-classical correlations such as: quantum discord, measurement-induced disturbance, measurement-induced nonlocality and geometric quantum discord^44–49^. It is worthwhile to mention that recently an increasing interest in quantum correlation measures based on skew information, namely local quantum uncertainty (LQU) and uncertainty-induced non-locality (UIN)^50–53^. Non-classical correlations between the NVD and external fields are one of the most extensively investigated subjects on the manipulation and storage of data^54–59^.

The organization of the paper is as follows: “The physical model” section involves the physical system and its mathematical model. In “NCC quantifiers” section the definition and the mathematical formula of the measures used to quantify the quantum correlations are presented. The reduced density matrices of the N-V centers as well as the reduced density matrix of the two nanocvities, which are used to analyze the dynamics of the non-classical correlations are introduced in “Dynamics of NCC quantifiers” section. The numerical results and the discussion are illustrated in “Numerical results and discussion” section. We summarize the results in “Conclusion” section.

## The physical model

Here, we consider a system consists of two open separated nanoscale photonic crystal cavities, each one contains coherently driven N-V center^58^. Each N-V center is a $\Lambda$-type three-level structure with the excited state $|A\rangle _{k} = (|E_{+}\rangle _{k}+ |E_{-}\rangle _{k})/ \sqrt{2} (k=A,B)$ as an ancillary state, where $|E_{\pm }\rangle _{k}$ are orbital states with angular momentum projection $\pm 1$ along the N-V axis. In the limit of low excitation, the $|A\rangle _{k}$ decays to the ground states $|0\rangle$ and $|1\rangle _{k}$. The photon-induced and laser-induced dynamic energy shifts are not considered due to the fact that the cavity is initially prepared in the vacuum state. In the dispersive regime, where the cavity mode is off-resonant with all transitions of the N-V centers and the PC-N-V coupling can be treated perturbatively, the effective interaction Hamiltonian between N-V center and nanocavity can then be written as^58,59^.

1 $$\begin{aligned} \hat{H}_{NV-C}=\tilde{g}\sum ^{2}_{i=1}(\hat{\psi }_{i}^{+}|g_{i}\rangle \langle e_{i}|+|e_{i}\rangle \langle g_{i}|\hat{\psi }_{i}), \end{aligned}$$

$\hat{\psi }_{i}^\dagger (\hat{\psi }_{i})$ refers to the creation (annihilation) operator for the effected quantized cavity fields and $\tilde{g}$ is the strength coupling between the N-V centers and the cavity field. $|e_{j}\rangle$ and $|g_{j}\rangle$ are the exited and ground states of the N-V centers. Next we consider the direct coupling of the two nanocavities, which is due to the finite overlap of their photonic wave functions with the following Hamiltonian,

2 $$\begin{aligned} \hat{H}_{h}= & {} -J(\hat{\psi }_{1}^{\dagger }\hat{\psi }_{2}+\hat{\psi }_{1}\hat{\psi }_{2}^{\dagger }), \end{aligned}$$

where *J* represents the hooping coupling constant between the two cavity fields, and can be considered as the distance between the two nanocavities inside the two photonic crystals.

If no photons exist in the systems, we consider the cavity field in the vacuum. The dissipative evolution of the system can be effectively represented using the non-Hermitian Hamiltonian,

3 $$\begin{aligned} \hat{H}_{d}= {} -\frac{i}{2}\sum ^{2}_{j=1}[\gamma _{j}\hat{\psi }_{j}^{\dagger }\hat{\psi }_{j}-\kappa _{j} |e_{j}\rangle \langle e_{j}|], \end{aligned}$$

where $\gamma _{j}$ is the decay rate of the *j*-cavity and $\kappa _{j}$ is the characteristic spontaneous decay rate from the N-V state $|e_{j}\rangle$ to the another state $|g_{j}\rangle$.

From Eqs. (1, 2, 3), the interaction picture of the total Hamiltonian can be written as:

4 $$\begin{aligned} \hat{H}=\hat{H}_{NV-C}+\hat{H}_{h}+\hat{H}_{d}. \end{aligned}$$

Following steps are to solve the system to find the density operator $\hat{\rho }(t)$, using the following formula:

5 $$\begin{aligned} i\hslash \frac{d}{dt}|\psi (t)\rangle =\,\hat{H}_{eff}\,|\psi (t)\rangle . \end{aligned}$$

Since the zero-excitation component is always invariant under the action of the effective non-Hermitian Hamiltonian, we can only consider the dynamics of the one-excitation subspace spanned by the basis vectors $$\{|0_{1} 0_{2} g_{1} g_{2}\rangle , |1_{1} 0_{2} g_{1} g_{2}\rangle , |0_{1} 1_{2} g_{1} g_{2}\rangle ,$$$$|0_{1} 0_{2} e_{1} g_{2}\rangle , |0_{1} 0_{2} g_{1} e_{2}\rangle \}$$, where $$|0_{j}\rangle$$ and $$|1_{j}\rangle$$ are the states of the *j*-cavity. The wave function of the final state of the system $$|\psi (t)\rangle$$ is represented by,

6 $$\begin{aligned} |\Psi (t)\rangle & = A(t)|1_{1}0_{2}g_{1}g_{2}\rangle +B(t)|0_{1}1_{2}g_{1}g_{2}\rangle +C(t)|0_{1}0_{2}e_{1}g_{2}\rangle \nonumber \\ &\quad +D(t)|0_{1}0_{2}g_{1}e_{2}\rangle +E(t)|0_{1}0_{2}g_{1}g_{2}\rangle . \end{aligned}$$

The parameters of the system state *A*(*t*), *B*(*t*), *C*(*t*) and *D*(*t*) are derived from Eq. (6) as

7 $$\begin{aligned} \dot{A}(t)= & {} -i\lambda _{1}C(t)+i J B(t)-\frac{\gamma _{1}}{2}A(t), \nonumber \\ \dot{B}(t)= & {} -i\lambda _{2}D(t)+i J A(t)-\frac{\gamma _{2}}{2}B(t), \nonumber \\ \dot{C}(t)= & {} -i\lambda _{1}A(t)-\frac{\kappa _{1}}{2}C(t), \nonumber \\ \dot{D}(t)= & {} -i\lambda _{2}B(t)-\frac{\kappa _{2}}{2}D(t). \end{aligned}$$

With $\dot{E}(t)=0$. The system of equations in (7) is numerically solved to find the final state of the system $|\psi (t)\rangle$ and its density matrix that is given by

8 $$\begin{aligned} \rho (t)=|\psi (t)\rangle \langle \psi (t)|, \end{aligned}$$

that is used to quantify the generated NCCs via the different quantifiers.

## NCC quantifiers

In this section, the definition of NCC quantifiers, which are based on the skew information quantity, the Bell function and the negativity, will be elucidated.

### Log-negativity entanglement

The log-negativity is a widely employed entanglement measure in quantum information theory, due to the fact that it is easy to compute and serves as an upper bound on distillable entanglement^60^. For a bipartite system $\rho ^{AB}$, the log-negativity *N*(*t*) is given by

9 $$\begin{aligned} N(t)= & {} \log _{2}[1+2 n(t)], \end{aligned}$$

where *n*(*t*) is the negativity and can be quantified by the absolute sum of the negative eigenvalues of the partial transpose matrix $(\rho ^{AB})^{T_{A}}$ with respect to subsystem *A*. The elements of density matrix $(\rho ^{AB})^{T_{A}}$ are given by,

10 $$\begin{aligned} \langle i, j|(\rho ^{AB})^{T_{A}}| m, n\rangle =\langle m, j|\rho ^{AB}| i, n\rangle . \end{aligned}$$

The value of *N*(*t*) defines the type of the system state where $N(t) =0$ for the separable states, and $N(t)\ne 0$ for entangled states.

### Skew information measures

The skew information quantity of a bipartite state $\rho ^{AB}$ is defined as

11 $$\begin{aligned} I(\rho ^{AB},K)=-\frac{1}{2}\text {Tr}\{[\sqrt{\rho ^{AB}},K\Bigr ]^{2}\}. \end{aligned}$$

This quantity is used as a measure of the information^50^ as well as uncertainty in a quantum state $\rho ^{AB}$ with respect to a local observable *K*. Based on the Skew information quantity, two NCC quantifiers local quantum uncertainty (LQU), and the uncertainty induced non-locality (UIN), were introduced.

#### Local quantum uncertainty LQU

Using the skew information quantity, LQU can be expressed as^61^:

12 $$\begin{aligned} LQU(\rho ^{AB})= & {} \min _{K}\{I(\rho ^{AB},K)\}. \end{aligned}$$

LQU quantifies the minimal quantum uncertainty in the system state $\rho ^{AB}$ over all the eigenvectors of the local observable *K*. For the two qubit state system that has $\rho ^{AB}(t)$, the LQU reduces to be^61^,

13 $$\begin{aligned} {L}(t)= & {} 1-\lambda _{max}(W_{AB}), \end{aligned}$$

where $\lambda _{max}$ is the largest eigenvalue of the $3 \times 3$-matrix $W_{AB}$ whose elements are given by,

14 $$\begin{aligned} w_{ij}=\text {T}r\big \{\sqrt{\rho ^{AB}(t)}(\sigma _{i}\otimes I)\sqrt{\rho ^{AB}(t)}(\sigma _{j}\otimes I)\big \}, \end{aligned}$$

and $\sigma _{i(j)},~i = 1,2,3$ are the Pauli operators,

#### Uncertainty induced non-locality

Similarly, based on the skew information quantity, the UIN can be defined using the following expression^53^,

15 $$\begin{aligned} UIN(\rho ^{AB})= & {} \max _{K} I(\rho ^{AB}(t),K). \end{aligned}$$

The $UIN(\rho ^{AB})$ is duel of $LOU(\rho ^{AB})$, and it can be defined by the maximal skew information of the state $\rho ^{AB}(t)$ and local observable *K*. As an update of the previous equation (15), it can be re-written as^53^:

16 $$\begin{aligned} U(t)= & {} \left\{ \begin{array}{ll} 1-\lambda _{\min }(W_{AB}), &{} {\vec {\mathbf{r}}=0;} \\ \\ 1-\frac{1}{\parallel \vec {\mathbf{r}}\parallel ^2} \vec {\mathbf{r}}\,\,W_{AB}\,\,\vec {\mathbf{r}}^{T}, &{} {\vec {\mathbf{r}}\ne 0}, \end{array} \right. \end{aligned}$$

The $\parallel \!\!\vec {\mathbf{r}}\!\!\parallel$ is the norm vector of the Bloch vector $\vec {\mathbf{r}}$.

### Bell function quantifier

Another measure that will be used in this paper to quantify the non-local correlation is the maximum Bell function MBF *M*(*t*)^35, 62^. As properties of the MBF, if $M(t)>2$, violation of the bell inequality occurs, which means that the non-classical correlation can be detected by MBF if it is greater than 2. The maximum Bell function for the state ($\rho ^{AB}$) can be expressed as follows:

17 $$\begin{aligned} M(t)= & {} 2 \sqrt{\xi _{1}+\xi _{2}}, \end{aligned}$$

where $\xi _{i} (i=1,2)$ are the two largest eigenvalues of the matrix ${T}^{\dagger }{T}$, and *T* is the correlation matrix^63^.

## Dynamics of NCC quantifiers

This section includes the reduced density matrices of the N-V centers as well as the reduced density matrix of the two nanocvities, which are used to analyze the dynamics of the non-classical correlations.

### Two N-V centers correlations

After applying several steps, to find the final state of the system of interaction, we extracted the reduced density matrix of the two N-V centers system, which can be written as:

18 $$\begin{aligned} \hat{\rho }_{N-V} (t)= & {} \text {Trace}_{Cav}\{\hat{\rho }(t)\}\nonumber \\= & {} \left( \begin{array}{cccc} \tilde{A}(t)\cdot \tilde{A}(t)^{*} &{} E(t)C(t)^{*} &{} E(t)D(t)^{*} &{} 0 \\ C(t)E(t)^{*} &{} C(t)C(t)^{*} &{} C(t)D(t)^{*} &{} 0 \\ D(t)E(t)^{*} &{} D(t)C(t)^{*} &{} D(t)D(t)^{*} &{} 0 \\ 0 &{} 0 &{} 0 &{} 0 \\ \end{array} \right) , \end{aligned}$$

where $\tilde{A}(t)\cdot \tilde{A}(t)^{*}=E(t)E(t)^{*}+A(t)A(t)^{*}+B(t)B(t)^{*}$, to make the parameter dimensionless, we set $\tilde{g}=1$, $\gamma _{i}=\kappa _{i}=\chi$. Here, we assume that the system initially starts with different cases, the uncorrelated and correlated state, as,

#### Uncorrelated state

First, we investigate the dynamics of non-classical correlation in case of the system state starts initially from the uncorrelated state $|\Psi (0)\rangle =|1_{1}0_{2}g_{1}g_{2}\rangle$. This to analyze the amount of the generated non-classical correlations and its robustness against the system parameters, coupling constants, spontaneous emission and decay rates.

#### Correlated state

Here, the system state initially starts with the correlated state,

$$\begin{aligned} |\Psi (0)\rangle =\frac{1}{\sqrt{2}}[|e_{1}g_{2}\rangle +|g_{1}e_{2}\rangle ]|0_{1}0_{2}\rangle , \end{aligned}$$

this to investigate the robustness of the generated correlations against the physical parameters.

### Two-nanocavity correlations

The reduced density matrix of the two-cavity system is given by

19 $$\begin{aligned} \hat{\rho }_{Cav}(t)= & {} \text {Trace}_{N-V}\{\hat{\rho }(t)\}\nonumber \\= & {} \left( \begin{array}{cccc} \tilde{C}(t)\tilde{C}(t)^{*} &{} E(t)A(t)^{*} &{} E(t)B(t)^{*} &{} 0 \\ A(t)E(t)^{*} &{} A(t)A(t)^{*} &{} A(t)B(t)^{*} &{} 0 \\ B(t)E(t)^{*} &{} B(t)A(t)^{*} &{} B(t)B(t)^{*} &{} 0 \\ 0 &{} 0 &{} 0 &{} 0 \\ \end{array} \right) , \end{aligned}$$

where $\tilde{C}(t)\cdot \tilde{C}(t)^{*}=E(t)E(t)^{*}+C(t)C(t)^{*}+D(t)D(t)^{*}$. In this paper, we investigate the correlation between the two cavities system when the initial state is an uncorrelated state $|\Psi (0)\rangle =|1_{1}0_{2}g_{1}g_{2}\rangle$. This to analyze the amount of the generated two-nanocavity correlations and its robustness against the spontaneous emission and decay rates.

## Numerical results and discussion

In this manuscript, we introduce a quantum open system consisting of two nitrogen-vacancy centers in two coupled nanocavities and leaking its photons to the external environment. We investigate the robustness of the generated non-classical correlations in the large coupling case $\tilde{g}\gg J$, the competition case $\tilde{g}=J$, and the large hopping case $\tilde{g} \ll J$ which are chosen according to a typical experiment^57,58^.

From the physics of this model, it is clear that the Hamiltonian consists of three parts: the first part represents the N-V centers in diamond and the connection between the centers and the cavity field is made through the coupling strength $\tilde{g}$, the second part is the two cavity fields whose interaction happens thought *J* “the cavity-cavity hopping strength”, and the third part of the Hamiltonian is the interaction part between the N-V centers and the cavity field. Another important parameter is $\chi$ that presents the dissipation rates of the spontaneous emission of the nitrogen-vacancy centers and the cavity dissipation. Thus, we can say that the relative interaction between the coupling strength $\tilde{g}$ and hopping *J* with value of decay rate $\chi$ has a great effect on the degree and dynamics of correlations over the system and consequently on the fidelity of the information transmission (quantum state transfer) between N-V centers. Hence, based on the system parameters, we explored three cases depending on the relation between the coupling strength $\tilde{g}$ and hopping interaction coupling *J*: the first case when $\tilde{g}\gg J$, means that the coupling strength between the N-V defects and the cavity field is grater than the cavity-cavity hopping interaction, the second case is the $\tilde{g} = J$, where the coupling between the N-V centers with the cavity field is equal to the hopping coupling interaction (it is known as competition case), and the third case is when the coupling strength between the N-V centers is less than the hopping coupling between the two cavities $\tilde{g} \ll J$. Moreover, we will investigate the effect of the cavity mode decay rate $\chi$.

**Figure 1:**
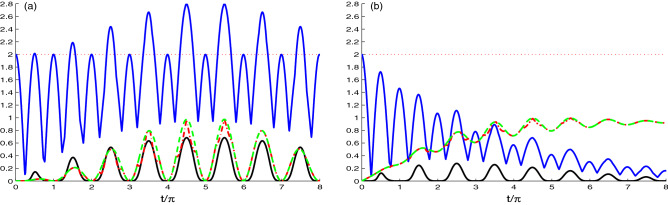
Time evolution of *L*(*t*) (dashed plots), *U*(*t*) (dashed dotted plots), *M*(*t*) (upper solid plots) and *N*(*t*) (solid plots) for large coupling case $J=0.1\tilde{g}$ with different decay rate $\chi =0.0$ in (**a**) and $\chi =0.1\tilde{g}$ in (**b**), where the NVD are prepared initially in uncorrelated state, $|\Psi (0)\rangle =|1_{1}0_{2}g_{1}g_{2}\rangle$.

**Figure 2:**
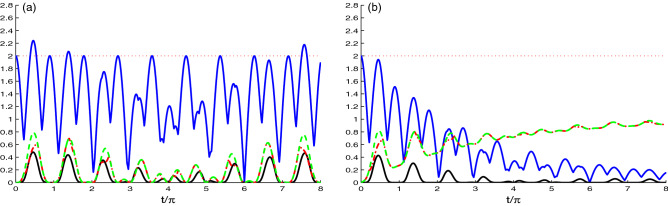
As Fig. 1 but for, the competition case $J=\tilde{g}$.

**Figure 3:**
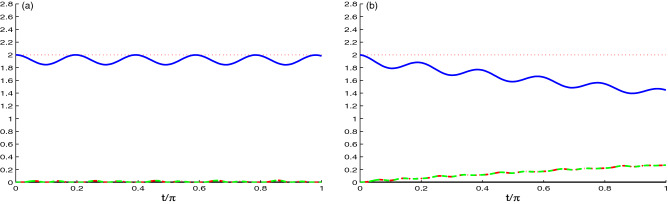
As Fig. 1 but for the case of the nancavities hopping coupling *J* greater than the N-V centers strength coupling $\tilde{g}$, where $J=10\tilde{g}$.

**Figure 4:**
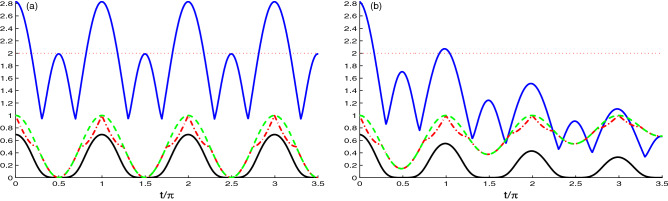
Time evolution of *L*(*t*) (dashed plots), *U*(*t*) (dashed dotted plots), *M*(*t*) (upper solid plots) and *N*(*t*) (solid plots) for large coupling case $J=0.1\tilde{g}$ with different decay rate $\chi =0.0$ in (**a**) and $\chi =0.1\tilde{g}$ in (**b**), where the NVD are prepared initially in correlated state, $|\Psi (0)\rangle =\frac{1}{\sqrt{2}}[|e_{1}g_{2}\rangle +|g_{1}e_{2}\rangle ]|0_{1}0_{2}\rangle$.

**Figure 5:**
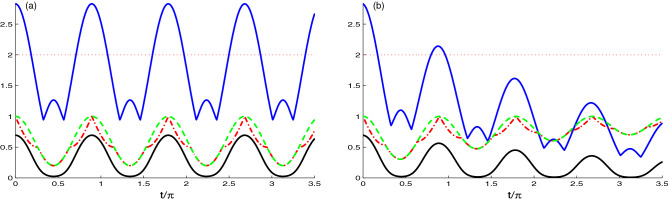
As Fig. 4 but for $J=\tilde{g}$.

**Figure 6:**
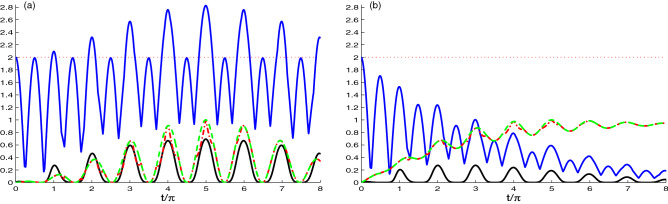
Time evolution of *L*(*t*) (dashed plots), *U*(*t*) (dashed dotted plots), *M*(*t*) (upper solid plots) and *N*(*t*) (solid plots) for $\hat{\rho }_{Cav}(t)$ and large coupling case $J=0.1\tilde{g}$ with different decay rate $\chi =0.0$ in (**a**) and $\chi =0.1\tilde{g}$ in (**b**).

**Figure 7:**
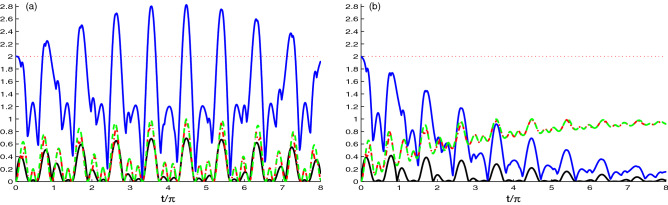
As Fig. 6 but for the competition case $J=\tilde{g}$.

**Figure 8:**
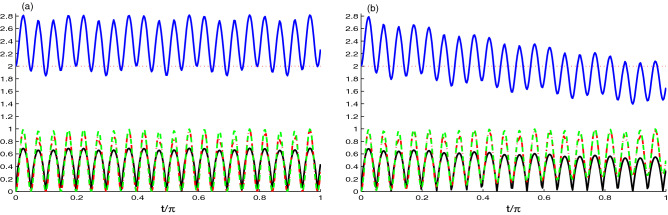
As Fig. 6 but for the case of the hopping coupling between the cavities grater than the coupling strength between the N-V centers, where $J=10\tilde{g}$.

Our numerical results are presented in Figs. 1, 2, 3, 4, 5, 6, 7, 8. Figure 1 illustrates the dynamics of the generated correlations between the NVD centers and quantified by skew information, Bell function and the log-negativity, where the dashed curve represents the dynamics of *L*(*t*), dashed-dotted curve displays the dynamics of *U*(*t*), upper solid curve represents the dynamics of *M*(*t*). Dynamics of *N*(*t*) is plotted as solid curve under the effect of the model parameters; strength coupling $\tilde{g}$, hopping coupling constant *J* and decay rate $\chi$, with nitrogen defects in diamond are prepared initially with an uncorrelated state $|\Psi (0)\rangle =|1_{1}0_{2}g_{1}g_{2}\rangle$, and the two cavities are in a vacuum state.

In Fig. 1a,b, we can see the dynamics of the non-classical correlations based on the first case where the NV-cavity coupling strength $\tilde{g}$ is greater than the hopping interaction strength ($J = 0.1\tilde{g}$) for different decay rates $\chi =0.0$ in (a) and $\chi =0.1\tilde{g}$ in (b). In Fig. 1a, we observe that NCC functions initially started from the minimum value $L(t) = U(t) = N(t) = 0$ (this is consistent with the fact that the two N-V centers started uncorrelated state) and with time increasing, the NCCs generate and increase with different oscillatory behaviors. The entanglement log-negativity measure *N*(*t*) “solid curve” increases more rapidly than the other measures where they reached $N(t) = 0.2$ at $t/\pi$ = 0.5, and the other measures reached the maximum value of the first oscillation $L(t) = U(t) = 0.3$ at $t/\pi$ = 1.5. Furthermore, it is clear that the values of $L(t) ~\text{ and } ~U(t)$ are equal and present the same behavior in some time intervals, where the LQU and UIN give the same correlation known as the skew-information correlation (SI correlation)^64^. It occurs at $L(t) = U(t))$ indicating that the minimal and maximal skew information correlations are equal. With the increase of time, we observe that all measures oscillate with a period of $t/\pi$ = 1. We can also note that in the third oscillation, there is a perfect match between all the value of measures. As the time evolves the maximum point of the oscillation (peak) increases and reaches the maximum value $L(t) = U(t) = 1$ at $t/\pi$ = 4.5 & 5.5, as the time furthermore, all the correlation vanish at $t/\pi =10$.

The upper solid curve in this Fig. 1a shows that the maximal violation of the Bell’s inequality ($M(t)>2$) appears in different intervals. In addition, this curve consists of 2 types of oscillations: The first one is with maximum value 2 and the other takes different values and reaches the maximum value $2\sqrt{2}$ at the same points of the maxima of the other measures. At these points, the unitary interaction is able to generate maximal NCCs between the two qubits.

Figure 1b indicates, the dynamics of the log-negativity, maximum Bell function, LQU and UIN correlations for the decay rate $\chi =0.1\tilde{g}$. The functions of the log-negativity, maximum Bell function correlations deteriorate and vanish completely after a particular time due to the coupling to the environment. While the LQU and UIN have the same behavior of the SI correlation, that attains asymptotically to its non-zero stationary value. This stationary skew-information follows the same behavior of the linear entropy as expected for the UIN^53^ of be an indicator to the mixedness.

Figure 2 is similar to Fig. 1, but for the N-V centers coupling strength equal to the cavity fields hooping coupling $J=\tilde{g}$. The change in the value of the coupling constants causes great change in the behavior of the NCCs. In Fig. 2a, the value of the hopping coupling constant is increased instead of the N-V centers strength coupling where $J=\tilde{g}$ and with zero decay rate $\chi =0.0$, but in Fig. 2b, $\chi =0.1\tilde{g}$. Figure 2a exhibits that increasing the hopping coupling constant *J*, gives an opposite effect compared with Fig. 1a, in which all measures started from the minimum value. Over time, the value of the measure increases and reaches the maximum value in the period $t/\pi \in$ [4.5–5.5].

Figure 2b shows the dynamics of all quantifiers under the effect of large values of the hopping coupling constant and large decay rate where $J=\chi =0.1\tilde{g}$. Compared to Fig. 1b, we find that all measures are effected by increasing the values of the hopping coupling constant and the decay rate. The oscillation of the correlation quantifiers $L(t), ~N(t) ~\text{ and } ~U(t)$ vanishes very fast compared to Fig. 1b. Moreover, the maximum value of $N(t) = 0.4$ at $\chi =0.5$ decays very fast. In this case, the generated NCCs in the systems is decreased due to the reduction of the strength coupling $\tilde{g}$. The correlation between the centers is generated due to the strength coupling between the N-V centers and the cavity.

Similar to Fig. 1, we investigated in Fig. 3 the dynamics of the non-classical correlations between the NVD centers, for the case where $J \gg \tilde{g}$, it means that the hopping coupling has larger contribution to the generated NCCs between the N-V centers than the strength NV-cavity coupling. Figure 3a reveals that the NCCs between the two N-V centers mainly result from the hopping two-cavity coupling, and small contribution is from the strength NV-cavity coupling, with vanishing decay rate $\chi =0.0$. The non-classical correlation between N-V centers, is more reduced compared to Fig. 1a. By decreasing the value of the coupling strength between the N-V centers, they become like separable and no correlation can be transferred between them. In addition, the three measures $L(t), ~N(t) ~\text{ and } ~U(t)$ are approximately vanishing. *M*(*t*) have a $\pi$-periodic behavior with decreasing amplitude. Figure 3b depicts the influence of the decay rate $\chi =0.1\tilde{g}$ on the dynamics of the correlation quantifiers. We observe, due to the decay rate, the skew information quantifiers increases only (dashed and dotted dashed curves), and reduces the Bell function quantifier *M*(*t*) (upper solid curve).

In Fig. 4, we use another initial state of the N-V centers in diamond, where $|\Psi (0)\rangle =\frac{1}{\sqrt{2}}[|e_{1}g_{2}\rangle +|g_{1}e_{2}\rangle ]|0_{1}0_{2}\rangle$ as the initial state of NVD. We use the same coupling constant and the decay rate values as in Fig. 1. In Fig. 4a, we use the coupling constant $J=0.1\tilde{g}$ for different decay rate. We observe that there is a difference between the dynamics of NCC measures in this figure and Fig. 1a. In this figure, the NCC measures start from its maximum value. Also, we can note that the dynamics of NCC quantifiers $L(t), ~N(t) ~\text{ and } ~U(t)$ have similar periodic oscillations. The measures $L(t)~\text{ and } ~U(t)$ are approximately the same, where they start from maximum value $L(t), ~N(t) ~\text{ and } ~U(t)$ = 1, and as the time evolves their values decreases and reach the minimum value $L(t), ~N(t) ~\text{ and } ~U(t) = 0$ at $t/\pi$ = 0.5. The dynamics of *M*(*t*) is periodic with with two types of oscillations: The first one oscillates between $M(t) = 2\sqrt{2}$ to 1, and the other one oscillates between $M(t)= 2$ to 1.

Figure 4b exhibits the effect of decay rate on the dynamics of entanglement. Based on the figure, we deduce that the Bell function correlation and the entanglement are very sensitive to the decay rate.

Figure 5a,b is the same as Fig. 4, but with increasing the value of hopping coupling constant $J=\tilde{g}$. It is clear that the NCC quantifiers $L(t), ~N(t) ~\text{ and } ~U(t)$ are not influenced by the change in the relative value of the hopping coupling constant and the strength coupling, they have similar dynamics as the ones in Fig. 4a,b. As we mentioned in Fig. 4 that the measure *M*(*t*) oscillates with two types of oscillation, here the small oscillation is affected. The maximum value is shifts to lower value and oscillates between 1 and 1.25. Fig. 5b reveal that all the correlations are almost insensitive to the ratio of the hopping constant to the coupling strength.

Figures 6, 7 shows the dynamics of the correlation generated between the two cavities. Figure 6 is displayed with the same parameters of the Fig. 1. In this figure, the strength coupling is greater than the hopping coupling ($J=0.1\tilde{g}$), so it plays the greatest role in generating the correlation between the two cavities. Figure 6a shows the behavior of the NCC quantifiers *L*(*t*), *U*(*t*) and *N*(*t*). It is clear that the behavior is the same as in Fig. 1a, where all started from zero, but the difference is that, the generation of correlation started later than in case of N-V centers in Fig. 1a. The second difference is the number of oscillations which is less than that in Fig. 1a. For Fig. 6b, it can be noted that the behaviour of the NCC takes the same behavior as in Fig. 1b, except the starting point of the correlation, where it started more later indicating that the decay rate has an observable effect on the dynamics of the NCC between the two cavities.

Figure 7 is the same as Fig. 6 but with the value of hopping coupling *J* equals to the value of the strength coupling $\tilde{g}$. We observe that the NCCs between the two cavity present similar behavior as the ones between the nitrogen vacancy centers (see Fig. 2). By increasing the hoping coupling see (Fig. 7a), of the NCCs are enhanced in amplitudes and frequency of the oscillations. The NCC quantifiers *L*(*t*), *U*(*t*) and *N*(*t*) start to be generated very fast. Comparing this figure and Fig. 2a, we observe that they have opposite behavior which is consistent with our expectations. The strength coupling is responsible for generating the NCCs between the N-V centers, while the hopping coupling is responsible for the correlations of the two cavities. Figure 7b shows the effect of increasing the hopping coupling $J=\tilde{g}$ and decay rate $\chi =0.1\tilde{g}$. These two parameters have opposite effect on the correlations. The hopping coupling enhances the generated correlations while the dissipation destroys them.

Figure 8 illustrates the correlations dynamics in the case of the hopping coupling between the two cavity fields higher than the coupling strength between the N-V centers in presence of the decay rate. In Fig. 8a, where we neglect the decay, we observe that by increasing the hopping coupling, the amplitude and the frequency of the correlation indicators are enhanced. Figure 8b depicts the effect of the decay rate $\chi =0.1\tilde{g}$. Also, two quantifiers *M*(*t*) (upper solid plots) and *N*(*t*) (solid plots) are reduced under the effect of the decay rate and the other two quantifiers, *L*(*t*) (dashed plots) and *U*(*t*) (dashed-dotted plots) are unchanged, which means that increasing the hopping coupling between the two cavities overcome the effect of the decay rate. Furthermore, we deduce that if the distance between the two cavities is very small, the decay rate has a small effect on the NCCs between the cavities.

From all figures, we observe that the hierarchy^64–67^ has been satisfied by the generated quantum correlations of the Bell function, the log-negativity and the skew-information quantifiers, meaning that Bell-nonlocality implies entanglement, which in turn implies LOU and UIN.

It is worth noting that the rigid hierarchy between the Bell nonlocality and the entanglement^64–67^ was proved and were reported in^65^. While the hierarchy between the negativity and the skew-information quantifiers is not reported explicitly. The skew-information quantifiers reduce to entanglement measure for uncorrelated states^53,61^. It is well known that the entanglement measures have ordering difficulties^68^.

The qualitative hierarchy (the existence and the amount of the generated correlation for the same state) depends on the two-cavity hooping coupling, the dissipation and the initial correlation. The qualitative hierarchy is achieved in the cases where (1) the hooping coupling is strong, (2) the system state initially starts with a maximally correlated state.

In some time intervals in Figs. 1 and 6, the hierarchy among the log-negativity and the skew-information quantifiers is only valid for the existence. In fact it is known that for the quantum quantifiers the hierarchy could be satisfied for the existence but not for the amount of the quantum correlations.

In the case of Figs. 1 and 6, the initial states are uncorrelated and the hooping coupling is very weak compared to the N-V centers and the cavity coupling, these explain that the hierarchy is only valid for the existence of the log-negativity and the skew information.

## Conclusion

In this paper, we have considered two Nitrogen Vacancies in Diamond (NVD) interacting with a cavity field. The N-V centers are prepared initially in the correlated and uncorrelated states. The model which simulates the interacting system is mathematically formulated and analytically solved. The generated non-classical correlations due to the interaction between the N-V centers and the two cavity fields are quantified using different measures based on the log-negativity, skew-information and Bell function. The sensitivity of the correlation dynamics to the effects of the initial state, couplings strength and decay rate are investigated. Our results showed that the initial state defines the shape of the generated NCCs. The decay rate has a destructive effect on all generated NCCs between the N-V centers. The coupling constants completely change the behavior of the correlation dynamics. For the strong hopping coupling only the non-classical correlations between the two nanocavities are generated. In addition, no notable NCCs are observed between the N-V centers. Finally, we deduce that the system parameters can be used as controllers for the dynamics of the generated non-classical correlations.
